# GABA_B_ Receptor Subunit GB1 at the Cell Surface Independently Activates ERK1/2 through IGF-1R Transactivation

**DOI:** 10.1371/journal.pone.0039698

**Published:** 2012-06-28

**Authors:** Guillaume A. Baloucoune, Lei Chun, Wenhua Zhang, Chanjuan Xu, Siluo Huang, Qian Sun, Yunyun Wang, Haijun Tu, Jianfeng Liu

**Affiliations:** Sino-France Laboratory for Drug Screening, Key Laboratory of Molecular Biophysics of Ministry of Education, College of Life Science and Technology, Huazhong University of Science and Technology, Wuhan, Hubei, China; University of Hong Kong, Hong Kong

## Abstract

**Background:**

Functional GABA_B_ receptor is believed to require hetero-dimerization between GABA_B1_ (GB1) and GABA_B2_ (GB2) subunits. The GB1 extracellular domain is required for ligand binding, and the GB2 trans-membrane domain is responsible for coupling to G proteins. Atypical GABA_B_ receptor responses observed in GB2-deficient mice suggested that GB1 may have activity in the absence of GB2. However the underlying mechanisms remain poorly characterized.

**Methodology/Principal Findings:**

Here, by using cells overexpressing a GB1 mutant (GB1asa) with the ability to translocate to the cell surface in the absence of GB2, we show that GABA_B_ receptor agonists, such as GABA and Baclofen, can induce ERK1/2 phosphorylation in the absence of GB2. Furthermore, we demonstrate that GB1asa induces ERK1/2 phosphorylation through Gi/o proteins and PLC dependent IGF-1R transactivation.

**Conclusions/Significance:**

Our data suggest that GB1 may form a functional receptor at the cell surface in the absence of GB2.

## Introduction

γ-aminobutyric acid (GABA) is a major inhibitory neurotransmitter in the mammalian central nervous system (CNS) [1] which mediates fast synaptic inhibition through ionotropic GABA_A_ and GABA_C_ receptors, as well as slow and prolonged synaptic inhibition through metabotropic GABA_B_ receptor [2]. The GABA_B_ receptor belongs to the class C G protein coupled receptors (GPCRs) and is composed of two distinct subunits, GABA_B1_ (GB1) and GABA_B2_ (GB2) [3]–[7]. The GB1 subunit binds to GABA, while the GB2 is responsible for the activation of Gi/o proteins [8], [9]. Evidence suggests that GB1 and GB2 hetero-dimerization is required for functional receptor formation. Cell surface trafficking of the GABA_B_ receptor is controlled by an endoplasmic reticulum (ER) retention signal (RSRR motif) in the intracellular C-terminus of the GB1 subunit. GB1 alone can’t translocate to the cell surface unless associated with GB2, through which the coil-coiled interaction between the C-terminus masks the ER retention signals [10], [11]. A mutation of the ER retention signal from RSRR to ASAR (GB1asa) allows GB1 to reach the cell surface independently [12].

During embryonic development, GB1 mRNA is detected in the hippocampal formation, cerebral cortex, intermediate and posterior neuroepithelium and the pontine neuroepithelium at E12, However, GB2 mRNA and protein are not detected at the same period in the central neuronal system (CNS) [13]. In adult organisms, whereas GB2 expression is limited to the brain [3], expression of GB1 is observed in most CNS regions and in peripheral tissues [1], [14]–[17]. GB1 and GB2 mRNAs are equally abundant in the cortex, thalamus, medial and lateral geniculate bodies, habenula, and cerebellum. Whereas the levels of GB2 mRNA are low to undetectable in caudater/putamen, hypothalamus, septum, preoptic area, and substantia nigra, GB1 mRNA is present at moderate to high levels. Likewise, whereas GB2 mRNA is undetectable in glial cells of white matter throughout the rat brain and spinal cord, expression of GB1 mRNA is detected in glial cells of all white matter and in glia throughout many areas of the brain [18]. These reports suggest that GB1 may be able to homodimerize, or heterodimerize with an unrecognized partner, and thus may exert GABA_B_ receptor-mediated physiological functions in the absence of GB2. Indeed, GB2-deficient (GB2^−/−^) mice show atypical electrophysiological GABA_B_ receptor responses in hippocampal slices [19]. However, in GB2-deficient mice whether GB1 exerts its function in the ER or at the cell surface, and how this effect is mediated, is not known.

In the present study, GB1asa-transfected HEK293 cells are used to address whether GB1asa at the cell surface is sufficient to induce ERK1/2 phosphorylation as a functional GABA_B_ receptor would do [20], [21]. We find that selective activation of GB1asa leads to ERK1/2 phosphorylation through Gi/o proteins and phospholipase C (PLC). Even more interestingly, we demonstrate that GB1asa-induced ERK1/2 phosphorylation occurs through transactivation of the IGF-1 receptor (IGF-1R).

## Materials and Methods

### Materials

GABA and IGF-1 were purchased from Sigma-Aldrich (St. Louis, MO, USA). Baclofen and CGP54626 were purchased from Tocris (Fisher-Bioblock, Illkrich, France). Pertussis toxin (PTX), U73122 and U73343 were purchased from Merck Biosciences (Darmstadt, Germany). Dulbecco’s modified Eagle’s medium (DMEM), penicillin, fetal bovine serum (FBS) and other solutions used for cell cultures were purchased from Invitrogen (Shanghai, China). PRK6 plasmids encoding wild-type GB1 and GB2 and mutant GB1asa with an epitope tag at their N-terminal ends under the control of a cytomegalovirus promoter were described previously [12]. Primary antibodies including phospho-p44/42 MAP kinase (T202/Y204) (# 9101) antibody, p44/42 MAPK (# 9102) antibody, phospho-Tyr1135/1136 IGF-IR antibody (19H7), IGF-IR antibody (111A9), were purchased from Cell Signaling Technology (Beverly, MA, USA). Anti-GB1 antibody (Ab55051) was purchased from Abcam (Cambridge, UK). Anti-GB2 (C-terminal) antibody was from Invitrogen (Shanghai, China). Anti-Flag-M2 monoclonal antibody was purchased from Sigma-Aldrich (St. Louis, MO, USA).

### Cell Culture and Transfection

Human embryonic kidney HEK293 cells were kindly provided by Dr. Philippe Rondard (Institut de Génomique Fonctionnelle, Montpellier, France) [21]. Mouse embryonic fibroblast (MEF) cells were kindly provided by Dr. Steve P. Balk (Beth Israel Deaconess Medical Center, Harvard Medical School, Boston, MA, USA) [22]. HEK293 cells were cultured in Dulbecco's modified Eagle's medium supplemented with 10% FBS and transfected by electroporation as described previously [23] or by lipofectamine 2000. For electroporation transfection, cells (10^7^) were transfected with plasmid DNA containing cDNAs encoding GB1asa (4 µg) or GB1 (4 µg) or GB2 (4 µg), with the addition of pRK6 vector to a total amount of 10 µg of plasmid DNA. For lipofectamine 2000 transfection, cells in 6-well plates were transfected with plasmid DNA containing cDNAs encoding GB1asa (500 ng) or GB1 (300 ng) or GB2 (500 ng), with the addition of pRK6 vector to a total amount of 800 ng of plasmid DNA.

### Quantification of Cell Surface Expression and Total Expression of GB1 by ELISA

After transfection with HA-tagged versions of the constructs by Lipofectamine 2000 according to the manufacturer’s protocol, HEK-293 cells were grown in DMEM at 37°C in a humidified atmosphere containing 95% air and 5% CO2 overnight and then split into white-walled, clear bottom 96-well plates coated with poly-L-lysine. 24 hrs later cells were washed twice with PBS, fixed with 4% paraformaldehyde in PBS (non-permeabilized for detection of thecell surface expression), or fixed with 4% paraformaldehyde and 0.1% Triton-100 in PBS (permeabilized for detection of the total expression), then blocked with PBS and 1% fetal calf serum.After 30 min incubation, the anti-HA monoclonal antibody conjugated with horseradish peroxidase (clone3F10, Roche Bioscience, Basel, Switzerland) was applied for 30 min and cells were washed. Bound antibody was detected by chemoluminescence using SuperSignal substrate (Pierce, Rockford, IL, USA) and a 2103 EnVision™ Multilabel Plate Readers (Perkin Elmer, Waltham, MA, USA).

### RNA Extraction and RT-PCR

24 hours after transfection, total RNA was extracted using Trizol and isolated according to the procedure supplied by the manufacturer (Invitrogen). Reverse transcription was carried out according to the manufacturer’s protocol (Invitrogen). The first strand of the cDNA was generated from 4 µg of total RNA using oligo-dT primer and M-MLV reverse transcriptase (Invitrogen). Nucleotide primers were prepared based on the sequences of human GB2 and β-actin. The sequences of these oligonucleotide primers were as follows: Primer for human GB2 (851 bp), 5′-ACCATCAGGTTCCAAGGATC-3′(forward) and 5′-AGGCAGAGGGTGATGGTGCT-3′ (reverse). Primer for human β-actin (290 bp): 5′-CGGAACCGCTCATTGCC-3′ (forward); antisense, 5′-ACCCACACTGTGCCCATCTA-3′ (reverse). The PCR was performed initially by denaturation at 94°C for 5 min, followed by 30 cycles of denaturation at 94°C for 1 min, annealing at 57°C for 1 min, extension at 72°C for 45 s (for GABA_B_R2, duration of 90 s), and a final extension step at 72°C for 10 min. Amplified DNA fragments were electrophoretically fractionated on 1% agarose gels.

### RNAi Transfection in Mouse Embryonic Fibroblast (MEF) Cells

IGF-R RNAi knockdown experiment using MEF cells were performed as previously described [24]. MEF cells were first transfected with shRNA and then with GB1asa (1 µg) by lipofectamine 2000. After 24 hrs, cells were treated with inhibitory compounds.

### Drug Treatments

Cultures were washed once with Ca^2+^-free HEPES-buffered solution (HBS) (containing 10 mM HEPES, pH 7.4, 140 mM NaCl, 4 mM KCl, 2 mM MgSO_4_ and 1 mM KH_2_PO_4_) and pre-incubated at 37°C with or without indicated inhibitors dissolved in HBS for 60 min. For PTX treatment, the cultures were pretreated for 14–16 hrs with PTX (200 ng/ml) or left untreated. Cells were then stimulated for the indicated time by incubating with GABA or IGF-1 prepared in fresh HBS. Inhibitors were dissolved in HBS with or without dimethyl sulfoxide (DMSO) or/and alcohol. Whenever DMSO or/and alcohol were used, HBS containing the same concentration of DMSO, alcohol, or both were used as the control vehicle. At the end of the treatment, the cells were quickly washed with ice-cold Ca^2+^-free PBS at pH 7.4, and 200 µl ice-cold lysis buffer (50 mM Tris pH 7.4, 150 mM NaCl, 1%SDS, 1 mM EDTA, 2.5 mM Sodium pyrophosphate, 1 mM β-glycerophosphate, 1 mM Na_3_VO_4_, 1 µg/ml leupeptin, 1 mM PMSF) was added to the cells and placed immediately on ice.

### Western Blot Analysis

Cell lysates were sonicated and protein concentrations were determined using Bradford reagent (Bio-Rad Laboratories Ltd., Hertfordshire, UK). Lysates were mixed with 4X SDS sample loading buffer (0.25 M Tris pH 6.8, 8% SDS, 40% glycerol, 0.4 M DTT, 0.04% bromophenol blue). Samples were boiled for 5 min then equal amounts of protein (20 µg) were resolved by SDS-PAGE on 8–12% gels. Proteins were transferred to nitrocellulose membranes (Millipore, Bedford, MA) and blocked in blocking buffer (5% nonfat dry milk in TBS and 0.1% Tween 20) for 1 hr at room temperature. The blots were then incubated with primer antibodies at the relevant dilution overnight at 4°C, and with horseradish peroxidase-linked secondary antibodies for 2 hrs. Immunoblots were detected using enhanced chemiluminescence reagents (Pierce Protein Research Products, Rockford, IL, USA) and visualized on X-ray film. The density of immunoreactive bands was measured by NIH imaging software, and all bands were normalized to percentages of control values.

**Figure 1:**
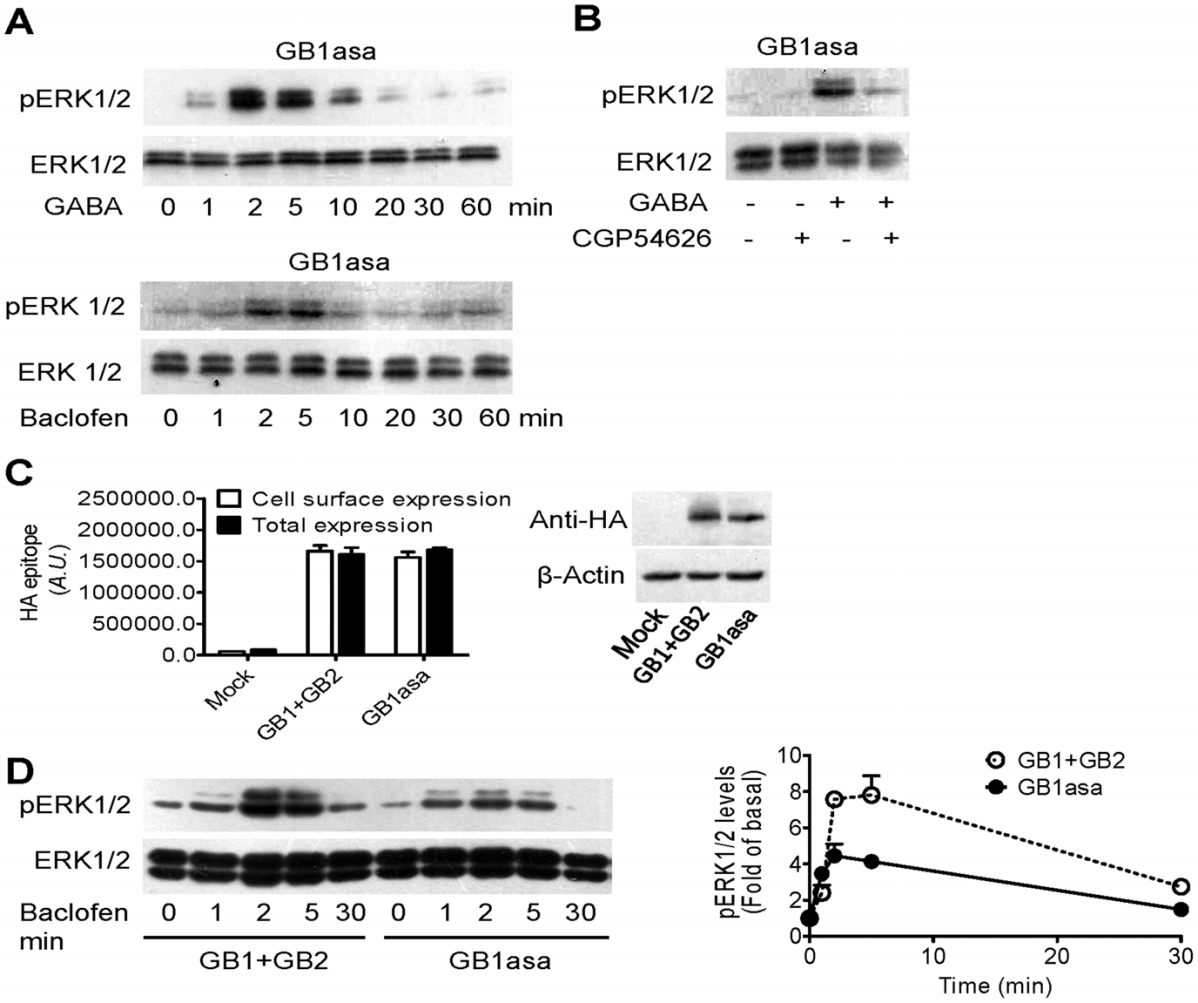
GB1asa can induce ERK1/2 phosphorylation independent of GB2. (A) Effects of GABA (100 µM) and Baclofen (100 µM) on ERK1/2 phosphorylation in cells overexpressing GB1asa over the indicated time course. (B) Effects of CGP54626 on GABA-induced ERK1/2 phosphorylation. CGP54626 (10 µM; 20 min) is incubated before treatment with GABA (100 µM; 3 min). (C) Detection of expression of ^HA^GB1asa alone or ^HA^GB1 in the presence of ^Flag^GB2 by ELISA (upper panel) and Western blots (lower panel). (D) Time course of the ERK1/2 phosphorylation induced by GABA (100 µM) in the HEK293 cells transfected with both GB1 and GB2 or GB1asa alone. The representative western blots are shown under the quantified data of ERK1/2 phosphorylation analyzed from at least three separate experiments (mean ± SEM).

### Statistical Analysis

Data are presented as means ± SEM of at least three independent experiments. Statistical analysis was performed by Student’s *t*-test. Values with *p*<0.05 were considered statistically significant.

**Figure 2:**
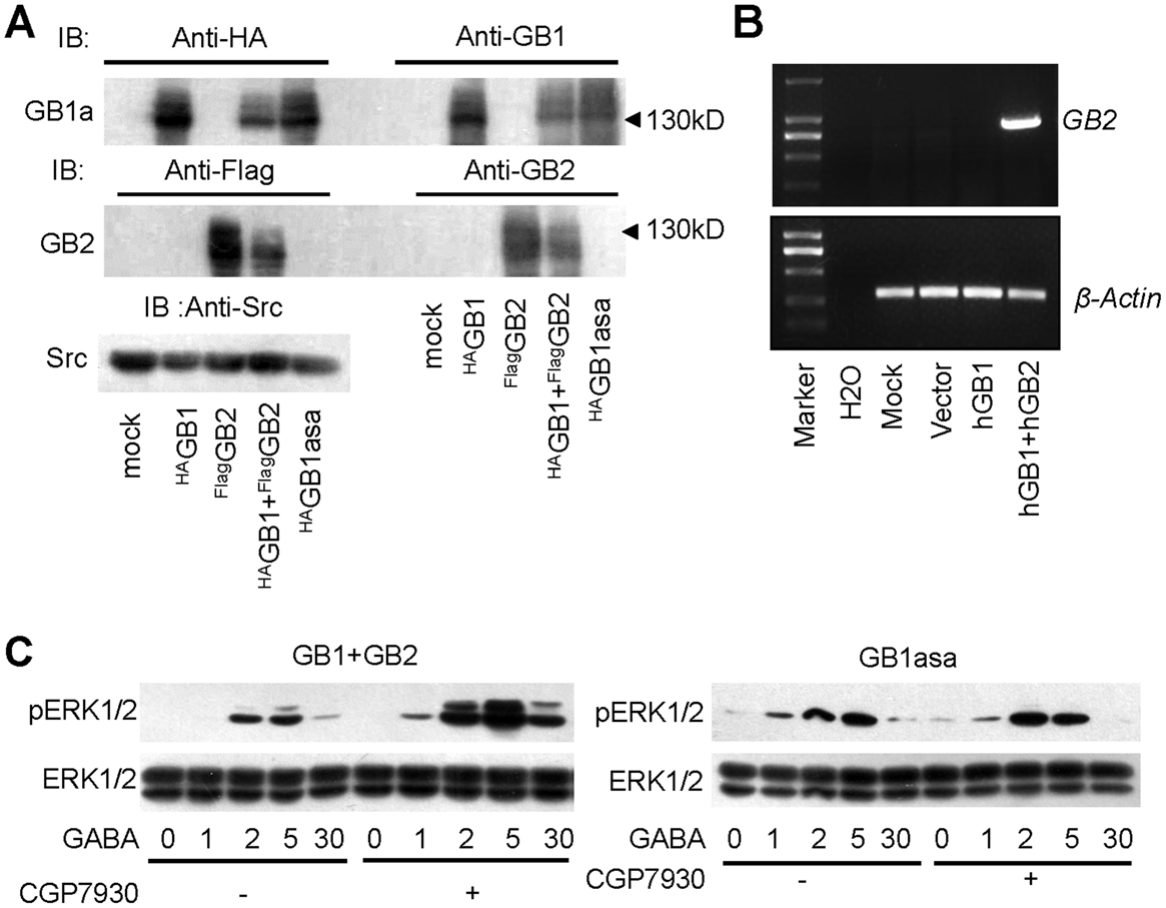
GB1asa-mediated ERK1/2 activation is independent of GABA_B2_. (A) Endogenous expression of GB2 is undetectable in HEK293 cells. Western blots of cell lysates performed with anti-HA or anti-GB1 antibodies (upper panel), anti-Flag or anti-GB2 antibodies (middle panel) and anti-Src antibody (bottom panel). Cells were transfected without or with ^HA^GB1, ^Flag^GB2, ^HA^GB1with ^Flag^GB2 and ^HA^GB1asa. (B) No GB2 mRNA can be detected in HEK293 cells. HEK293 cells transfected with human GB1 or human GB1 with human GB2 are used as negative and positive control respectively. Images are representative of at least three independent RT-PCR analyses. (C) Time course of the ERK1/2 phosphorylation induced by GABA (100 µM) in the absence or presence of CGP7930 (25 µM) in the HEK293 cells transfected with both GB1 and GB2 (left panel) or GB1asa alone (right panel). All western blots shown here are representative of at least three separate experiments.

## Results

### GB1asa can Induce ERK1/2 Phosphorylation Independent of GB2

Functional heterodimeric GABA_B_ receptor induces ERK1/2 phosphorylation in neurons [20], [21]. To test whether GB1 can activate ERK1/2 phosphorylation in the absence of GB2, HEK293 cells were transfected with a GB1 mutant, GB1asa. This mutant is able to translocate to the cell surface independent of GB2. Specific GABA_B_ receptor agonists, such as GABA (100 µM) or Baclofen (100 µM), induced ERK1/2 phosphorylation in a transient manner in cells overexpressing only GB1asa (**Fig. 1A**). We further evaluated the effect of the GABA_B_ receptor-selective antagonist, CGP54626, on GABA-induced ERK1/2 phosphorylation. CGP54626 pretreatment (10 µM) blocked GABA (100 µM)-induced ERK1/2 phosphorylation (**Fig. 1B**), thereby demonstrating that selective activation of GB1asa induced ERK1/2 phosphorylation.

**Figure 3:**
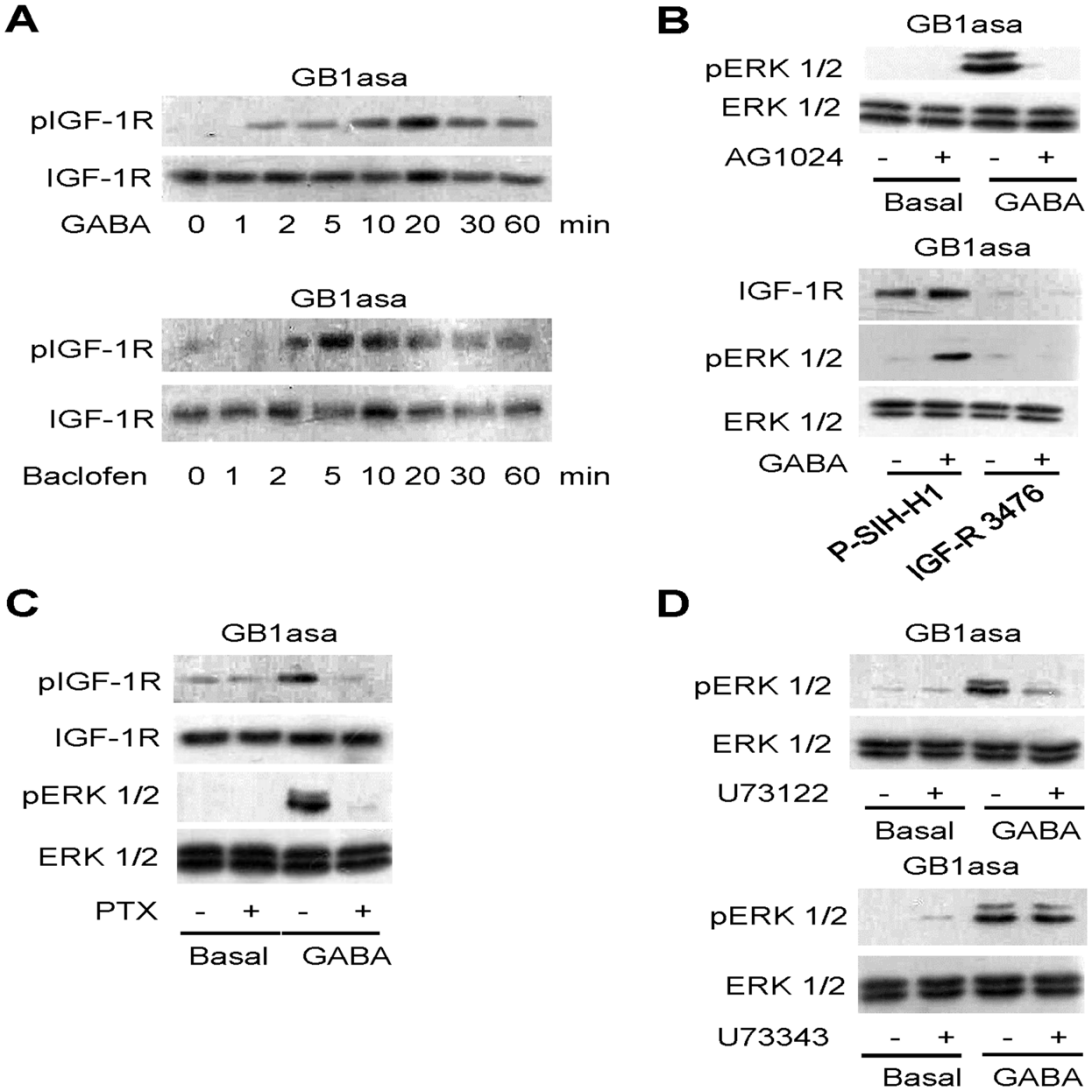
GB1asa-mediated ERK1/2 phosphorylation requires IGF-1R transactivation through Gi/o proteins and PLC pathway. (A) Effects of GABA (100 µM) and Baclofen (100 µM) on IGF-1R phosphorylation in cells overexpressing GB1asa for the indicated time course. (B) Effect of AG1024 (upper panel) and shRNA of IGF-1R (3476) (lower panel) on GABA-stimulated ERK1/2 phosphorylation. AG1024 (0.1 µM; 60 min) is incubated before treatment with GABA (100 µM; 5 min) in HEK293 cells overexpressing GB1asa. The shRNA knock-down assay is performed in MEF cells overexpressing GB1asa. (C) Effect of PTX on GABA-stimulated IGF-1R and ERK1/2 phosphorylation. PTX (200 ng/ml; 16 hrs) is incubated before and during treatment with GABA (100 µM; 5 min). (D) Effect of U73122 and U73343 on GABA-stimulated ERK1/2 phosphorylation. U73122 (5 µM; 60 min) or U73343 (5 µM; 60 min) are incubated before treatment with GABA (100 µM; 5 min). The western blots shown are representative of at least three separate experiments.

To investigate whether GB1asa allows phosphorylation of EKR1/2 with similar efficiency as heterodimeric GABA_B_ receptor, we expressed either GB1asa, or GB1 wild type (GB1) and GB2, at the cell surface with comparable expression levels (**Fig 1C**). In these assays, all transfected GB1asa trafficked to the cell surface (**Fig 1C**) and baclofen-induced ERK1/2 phosphorylation was higher in cells co-expressing both GB1 and GB2 than that of cells overexpressing GB1asa (**Fig 1D**), suggesting that though GB1asa may form a functional receptor at the cell surface in the absence of GB2, its efficacy was less than heterodimeric GABA_B_ receptor.

**Figure 4:**
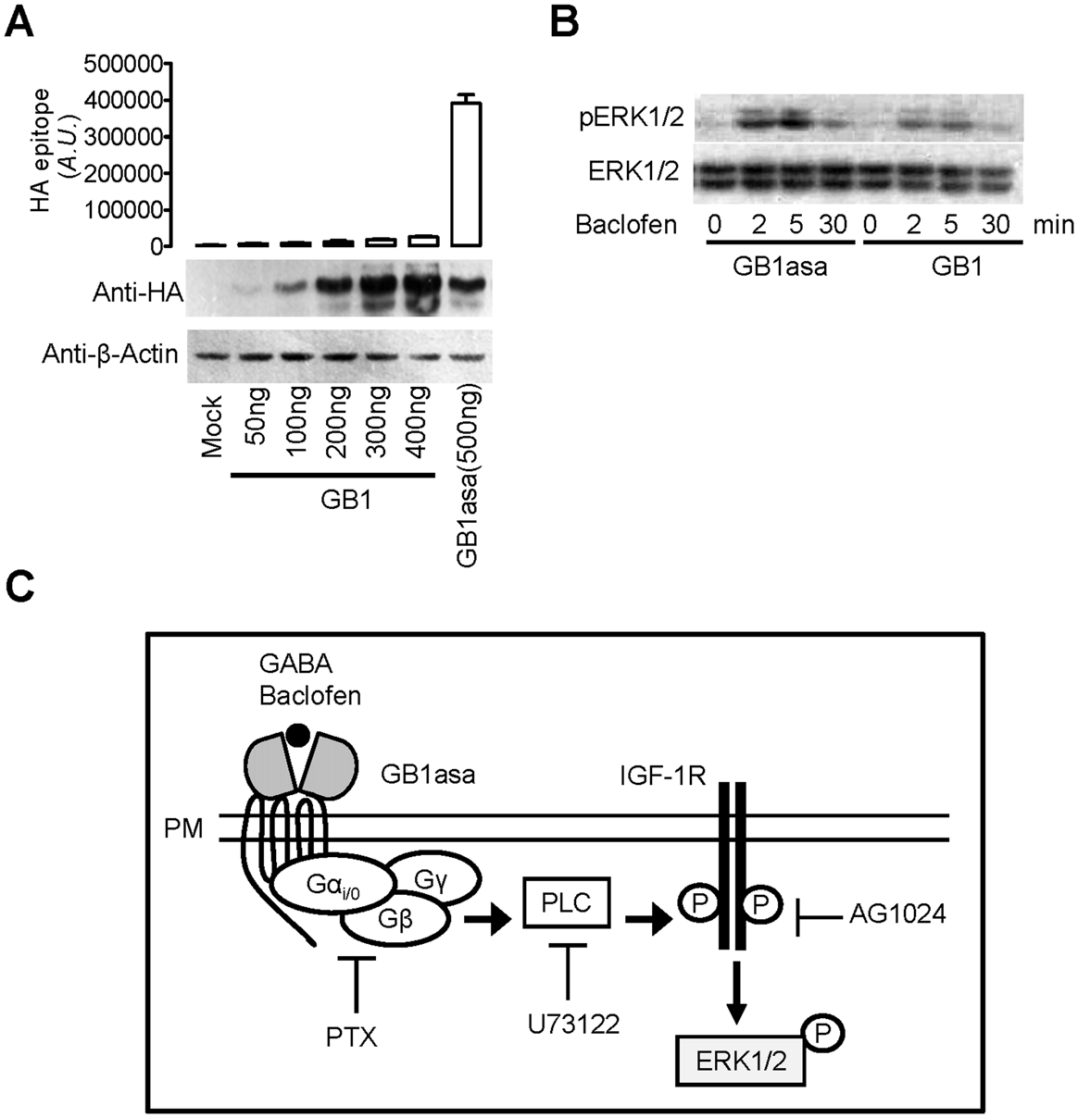
In the absence of GB2, GB1asa induction of ERK1/2 phosphorylation is greater than induction by wild type GB1. (A) Detection of the cell surface expression of GB1 or GB1asa (upper panel) and total expression by Western blots with anti-HA and anti-β-actin (lower panel). (B) Time course of the endogenous ERK1/2 phosphorylation induced by GABA (100 µM) in the HEK293 cells transfected with GB1asa or GB1 alone. (C) Schematic representation of the signaling pathway mediated by GB1asa at the cell surface. Activation of ERK1/2 phosphorylation by GB1asa requires Gi/o proteins to activate PLC pathway, which in turn transactivates IGF-IR.

We have shown previously that GABA_B_ receptor induced ERK1/2 phosphorylation through GB2 subunits [21]. We therefore tested whether GB1asa-mediated ERK1/2 phosphorylation is due to endogenous GB2. By using antibodies against the C-terminus of GB1 and GB2 or antibodies against the epitope tag fused to the N-terminals of GB1 and GB2, we found that no endogenous GB1 or GB2 was detectable in HEK293 cells (**Fig. 2A**). Furthermore, we also failed to detect any GB2 mRNA expression in HEK293 cells (**Fig. 2B**). It has been shown that a positive allosteric modulator, CGP7930, can increase GABA_B_ receptor downstream signaling through binding to GB2 subunits trans-membrane domains [21], [25]. Indeed, we found that whereas CGP7930 increases GABA-induced ERK1/2 phosphorylation in cells overexpressing both GB1 and GB2, CGP7930 failed to do so in cells expressing GB1asa alone (**Fig. 2C**), further demonstrating that GB1asa induced ERK1/2 phosphorylation via a GB2-independent pathway.

### GB1asa Induces ERK1/2 Phosphorylation via IGF-1R Transactivation and Gi/o-protein/PLC pathway

We next examined how GB1asa induced ERK1/2 phosphorylation. We have recently reported that GABA_B_ receptor protected neurons from apoptosis via IGF-1R transactivation [22]. Here, we investigated whether IGF-1R mediated the phosphorylation of ERK1/2 induced by GB1asa. Both GABA (100 µM) and baclofen (100 µM) induced IGF-1R phosphorylation in a transient manner without altering IGF-1R protein expression in GB1asa-tranfected HEK293 cells (**Fig. 3A**). We further performed the experiments with either a pharmacological inhibitor, AG1024 (a specific inhibitor of IGF-IR phosphorylation), or RNAi knock-down approaches. Indeed, AG1024 pretreatment inhibited GABA-induced ERK1/2 phosphorylation in GB1asa-transfected HEK293 cells (**Fig. 3B** **upper panel**). Furthermore, transfection of IGF-1R RNAi (IGF-R3467) also inhibited GABA-induced ERK1/2 phosphorylation by reducing endogenous IGF-1R expression in GB1asa-transfected MEF cells; whereas, scrambled RNAi (P-SIH-H1) had no such effect (**Fig. 3B** **lower panel**). In all, these results demonstrated that GB1asa induced ERK1/2 phosphorylation through IGF-1R transactivation.

We have previously shown that GABA_B_ receptor transactivated IGF-1R through Gi/o proteins [22]. Pertussis toxin (PTX) pretreatment (200 ng/ml) abolished GB1asa-induced IGF-1R and ERK1/2 phosphorylation (**Fig. 3C**), suggesting that Gi/o proteins are involved in GB1asa-mediated IGF-1R transactivation, which in turn induces ERK1/2 phosphorylation.

Functional GABA_B_ receptor has also been shown to enhance phospholipase C (PLC) activity through the Gβγ subunits [22]. We examined the possible involvement of PLC on GB1asa-induced ERK1/2 phosphorylation. We found that pretreating cells with U73122, an inhibitor of PLC, but not its inactive analog U73343, completely abolished ERK1/2 phosphorylation (**Fig. 3D**), thus suggesting that GB1asa-induced ERK1/2 activation is mediated through PLC.

### In the Absence of GB2, GB1asa Induction of ERK1/2 Phosphorylation is Greater than Induction by Wild type GB1

It has been shown that intracellular GB1 alone induces ERK1/2 phosphorylation [26]. Here we compared the effect induced either by GB1 or GB1asa. We expressed either GB1asa, or GB1 wild type (GB1) with comparable expression levels, whereas only GB1asa could be expressed at the cell surface (**Fig 4A**). Under these conditions, GB1asa-induced ERK1/2 phosphorylation was much higher than GB1-induced ERK1/2 phosphorylation (**Fig 4B**), suggesting that cell surface located GB1 more efficiently induces ERK1/2 phosphorylation than intracellular GB1.

## Discussion

In the present study, we demonstrated that a GB1 mutant, GB1asa, was able to act at the cell surface to induce ERK1/2 phosphorylation in a manner independent of GB2. Furthermore, we found that GB1asa-induced ERK1/2 phosphorylation acts via Gi/o-proteins and the PLC-mediated IGF-1R transactivation (**Fig 4C**).

Hetero-dimerization is a prerequisite for native GABA_B_ receptor function. GB2 masks the ER retention signal located at the C-terminus of GB1, thereby allowing GB1 to reach the cell surface [2], [10]–[12]. However, the temporal and spatial expression profiles of GB1 and GB2 do not always coincide [1], [3], [13], [18], suggesting that GB1 is functional in the absence of GB2. Furthermore, several lines of evidence suggest that GB1, independent of GB2, interacts with Kir3.1 channels [27], induces ERK1/2 phosphorylation and regulates leptin mRNA expression [26], [28]. However, all of these reports failed to detect obvious cell surface expression of GB1 in the absence of GB2. It is likely that GB1 alone has activity, possibly as a homodimer on the ER and ER-Golgi intermediate compartment [29]. However, it remains unclear how ligands can enter the cell to induce a rapid response through intracellular receptors. To circumvent these issues, we use the ER retention signal mutant of GB1, GB1asa, which can translocate to the cell surface independently of GB2, to show that GB1asa at the cell surface can act as a functional receptor to induce ERK1/2 phosphorylation. Furthermore, GB1asa-induced ERK1/2 phosphorylation is much higher than mediated by transfected wild type GB1, suggesting that cell surface expression of GB1 allows for more efficient ERK1/2 activation. How intracellular GB1 induces ERK1/2 phosphorylation remains for further investigation.

Even though GB1asa utilizes an artificial mechanism to allow translocation to the plasma membrane, it is probable that GB1 can be trafficked to the cell surface in the absence of GB2. It has been shown that a novel GPCR interacting scaffolding protein (GISP) can facilitate the transportation of GB1 to the cell surface by direct interaction with the coiled-coil domain of GB1 C-terminus, thus allowing translocation of GB1 independent of GB2 [30]. Furthermore, association of GB1 with the GABA_A_ receptor γ2S subunit promotes GB1 cell surface expression in the absence of GB2 [31]. Further efforts need to be devoted to elucidating the mechanisms that allow traffic of GB1 towards the cell surface in the absence of GB2.

Our data shows that GB1asa at the cell surface in the absence of GB2 is sufficient to activate ERK1/2 via Gi/o proteins and PLC pathway, though with less efficiency than in the presence of GB2. Gi/o proteins are pre-associated with GABA_B_ receptor [32], [33]. GABA_B_ receptor transactivates IGF-1R through Gi/o protein βγ subunits, which in turn activates the PLC pathway [22]. βγ subunits of G proteins produced by GABA_B_ receptor enhance the mGluR-mediated Gq response [34]. It is possible that preassembled Gi/o proteins interact with GB1. Upon activation, GB1 could induce Gi/o proteins βγ subunits to transactivate IGF-1R via the PLC pathway. Although no Gi/o proteins coupled to GB1 in the absence of GB2 has been previously detected [8], [9], [35], [36], our results suggest that GB1 retains the ability to couple Gi/o proteins in the absence of GB2, but with decreased efficiency. Whether and how GB1alone interacts with Gi/o proteins needs to be further characterized.

In summary, our data demonstrate that a plasma membrane localized GB1 mutant, GB1asa, induced ERK1/2 phosphorylation through Gi/o proteins and PLC-dependent transactivation of IGF-1R. These results provide the first evidence that GB1 at the cell surface may exert the same function as heterodimeric GABA_B_ receptor, suggesting a novel mechanism of activation by this receptor.
